# Endometrial microbiota alteration in female patients with endometrial polyps based on 16S rRNA gene sequencing analysis

**DOI:** 10.3389/fcimb.2024.1351329

**Published:** 2024-04-09

**Authors:** Yu Zhao, Yun Liao, Gufeng Xu, Yue Wang

**Affiliations:** ^1^ Department of Ambulatory Surgery, Women’s Hospital School of Medicine Zhejiang University, Hangzhou, China; ^2^ Zhejiang Provincial Clinical Research Center for Obstetrics and Gynecology, Hangzhou, China; ^3^ Zhejiang Provincial Key Laboratory of Precision Diagnosis and Therapy for Major Gynecological Diseases, Women’s Hospital School of Medicine Zhejiang University, Hangzhou, China

**Keywords:** endometrial polyp, endometrial microbiota, 16S rRNA, biomarker, predictive model

## Abstract

**Introduction:**

The potential role of the endometrial microbiota in the pathogenesis of endometrial polyps (EPs) warrants further investigation, given the current landscape of limited and inconclusive research findings. We aimed to explore the microecological characteristics of the uterine cavity in patients with EPs and investigate the potential of endometrial microbiota species as novel biomarkers for identifying EPs.

**Methods:**

Endometrial samples were collected from 225 patients who underwent hysteroscopies, of whom 167 had EPs, whereas 58 had non- hyperproliferative endometrium status. The endometrial microbiota was assessed using 16S rRNA gene sequencing. We characterized the endometrial microbiota and identified microbial biomarkers for predicting EPs.

**Results:**

The endometrial microbial diversity and composition were significantly different between the EP and control groups. Predictive functional analyses of the endometrial microbiota demonstrated significant alterations in pathways involved in sphingolipid metabolism, steroid hormone biosynthesis, and apoptosis between the two groups. Moreover, a classification model based on endometrial microbial ASV-based biomarkers along with the presence of abnormal uterine bleeding symptoms achieved powerful classification potential in identifying EPs in both the discovery and validation cohorts.

**Conclusion:**

Our study indicates a potential association between altered endometrial microbiota and EPs. Endometrial microbiota-based biomarkers may prove valuable for the diagnosis of EPs.

**Clinical trial registration:**

Chinese Clinical Trial Registry (ChiCTR2100052746).

## 1 Introduction

Endometrial polyps (EPs) are protrusions that develop in the uterine cavity due to abnormal growth of the glands, stroma, and blood vessels of the endometrium. EP is a common benign uterine condition, with reported incidence rates ranging from 8% to 35% in both premenopausal and postmenopausal women (American Association of Gynecologic, 2012). EP can lead to symptoms such as abnormal uterine bleeding and infertility. EP occurs in approximately 30% of infertile women (Munro, 2019) and nearly 28% of cases involving postmenopausal bleeding (Nijkang et al., 2019). Currently, reliable and effective pharmacological treatments for EP are lacking. Hysteroscopic polypectomy is the most effective surgical intervention for EP; however, it is associated with a high rate of postoperative recurrence, ranging from 3% to 46%.

To date, the exact cause of EP is not fully understood, but recent studies have elucidated various factors that may contribute to the development of EP. These factors include imbalances in hormones, abnormal proliferation of endometrial cells, disrupted apoptosis, activation of vascular endothelial growth factor leading to the formation of thick-walled blood vessels, and immune and metabolic disorders. Emerging research suggests a potential connection between immune-inflammatory reactions, chronic endometritis, and EP onset. A meta-analysis conducted on premenopausal women with EP who often experienced abnormal uterine bleeding (AUB) revealed a significant association between chronic endometritis and EP. The presence of CD138+ plasma cells (Vitagliano et al., 2021), which are markers of chronic inflammation, coincides with premenopausal EP, indicating a possible link between inflammation and EP development. Furthermore, analysis of the vaginal microbiota in patients with EP compared to that in healthy women revealed a significant increase in the abundance and diversity of microbiota in the vaginas of patients with EP (Kim et al., 2022). These findings suggest a potential correlation among microbial imbalance, inflammation, and endometrial pathology in relation to EP.

Maintaining a balanced microbiota in the female reproductive tract is crucial for reproductive health. Recent advancements in second-generation sequencing have provided evidence that the microbiota present in the endometrium is unique, complex, and diverse compared with the microbial composition of the vagina and cervix. In 2016, Fang et al. utilized barcoded sequencing technology to investigate the microbial composition of the uterine cavity in patients with EP. The study revealed that patients with EP had a higher abundance of *Lactobacillus*, *Bifidobacterium*, *Gardnerella*, *Streptococcus*, *Altermonas*, and *Prevotella*, whereas the proportions of *Fusobacterium*, *Pseudomonas*, *Escherichia coli*, and *Sphingomonas* were lower than those in healthy women (Fang et al., 2016). Subsequent studies have also reported that the microbiota of the endometrium in patients with EP primarily consisted of anaerobic bacteria, such as *Bacteroides* and *Fusobacterium*, as well as aerobic bacteria, such as *Staphylococcus* and *Streptococcus* (Horban et al., 2019; Liang et al., 2023). However, further verification is required to ensure consistency in these results. In addition, investigating the potential functional changes of microbiota and their interactions with the host is currently a prominent area of research in reproductive tract microbiota studies. Previous studies have indicated potential functional alterations in the endometrial microbiota of patients with endometriosis and endometrial cancer (Knapp et al., 2010; Lee et al., 2014), particularly in pathways such as sphingolipid metabolism. However, these alterations have not been explored in studies focusing on EPs. Meanwhile, due to limitations in sample size, the aforementioned studies were unable to conduct further analyses to explore the predictive value of microbial-based indicators for diagnosing EPs.

This study used 16S ribosomal RNA (16S rRNA) gene sequencing technology to investigate and analyze the microbiota present in endometrial samples obtained from patients diagnosed with EP or other uterine disorders. The primary objective of this study was to further investigate the microbial characteristics of the uterine cavity in patients with EP by comparing them to individuals without endometrial hyperproliferative diseases and to examine the possible role of endometrial microbiota as novel biomarkers for identifying EPs.

## 2 Materials and methods

### 2.1 Study design and patient selection

This study aimed to characterize the endometrial microbiota in patients diagnosed with EPs using 16S rRNA gene sequencing. The study enrolled patients diagnosed with uterine diseases who underwent hysteroscopic surgery at the Women’s Hospital, School of Medicine, Zhejiang University, between March 2022 and December 2022. Inclusion criteria included: (1) an age range of 18 to 55 years and (2) premenopausal status. Exclusion criteria encompassed: (1) acute stage of any female genital tract inflammatory disease; (2) pregnancy; and (3) active HPV infection or detectable anomalies in cervical Thinprep Cytologic Test (TCT) results. To ensure the validity of the study, all patients were confirmed not to have used any oral or vaginal antibiotics within the month preceding surgery and were also required to have abstained from sexual activity following their last menstruation. The study has been approved by the Ethics Committee of the Women’s Hospital, School of Medicine, Zhejiang University (IRB-20210280-R), and written informed consent was obtained from all participants.

### 2.2 Sample collection and hysteroscopic examination

Using hysteroscopic surgery, endometrial specimens were collected during the follicular phase following menstruation. Standard sterilization procedures were performed on the vagina and cervix after the administration of intravenous anesthesia. A sterile vaginal speculum was inserted into the vagina to expose the cervix. A soft-pipeline endometrial biopsy catheter (Qiuheng Medical Devices, China) was then inserted into the uterine cavity through the cervical canal to obtain endometrial samples. Visual control was maintained throughout the procedure to prevent contact with the vaginal wall. After the collection of the samples, they were immediately transferred into sterile cryogenic storage tubes and stored in a refrigerator at -80 °C for subsequent analysis.

After sample collection, uterine manifestations were documented using hysteroscopy. This examination included recording details, such as the type of lesions present and their quantity, size, and location within the uterus.

### 2.3 Patient grouping and pathological assessment

Patients participating in the study were grouped based on their hysteroscopic diagnosis and histopathological examination results. Patients diagnosed with endometrial polyps through a combination of hysteroscopic examination and pathological confirmation were classified into the EP group. Patients who did not exhibit any signs of endometrial polyps were included in the control group. Patients with other endometrial hyperproliferative diseases, such as endometrial hyperplasia with or without atypical hyperplasia or endometrial cancer, were excluded from the study. Baseline clinical data, including age, gravidity (number of pregnancies), parity (number of live births), symptoms, and surgical history, were recorded for all participants. To evaluate chronic endometritis (CE), CD138 immunohistochemical (IHC) staining was performed simultaneously with endometrial pathology. In brief, mouse monoclonal antibody clone GR106 against human Syndecan-1 (Gene Technology, Shanghai) was incubated on slides for an entire night at 4°C. The secondary rabbit anti-mouse horseradish peroxidase-labeled antibody ab97046 (Abcam, UK) was then applied to the slides and incubated for an additional hour. The cell was regarded to be a CD138+ plasma cell if it displayed complete, unambiguous, brown staining with intact cell membranes. They were counted under a light microscope (400-fold magnification, a high-powered field; HPF). The diagnosis of CE was based on the presence of one or more CD138+ plasma cells in 10 non-overlapping random stromal areas.

### 2.4 Genomic DNA extraction and 16S ribosomal RNA gene sequencing

To extract genomic DNA from endometrial samples, the cetyltrimethylammonium bromide (CTAB) method was employed, following the protocol which has been described in detail previously (Huang et al., 2018). Nuclear-free water was used as the negative control (blank) during DNA extraction. DNA integrity and size were verified using 1.0% agarose gel electrophoresis, and DNA concentrations were quantified using a NanoDrop spectrophotometer (NanoDrop, Germany).

For the analysis of the V3 and V4 regions of the 16S ribosomal RNA (rRNA) gene, universal primers 341F (5’-CCTACGGGNGGCWGCAG-3’) and 805R (5’-GACTACHVGGGTATCTAATCC-3’) were used to amplify the specific regions. To differentiate each sample and yield accurate phylogenetic and taxonomic information, the gene products were attached with forward and reverse error-correcting barcodes. PCR amplification was performed to target these regions for subsequent analysis. The resulting PCR products were purified using AMPure XT beads (Beckman Coulter Genomics, USA) and quantified using a Qubit fluorometer (Invitrogen, USA). Subsequently, amplicon pools were prepared for sequencing. The size and quantity of the amplicon library were assessed using an Agilent 2100 Bioanalyzer (Agilent, USA) and the Library Quantification Kit from Illumina (Kapa Biosciences, USA), respectively. Finally, the libraries were sequenced on a NovaSeq PE250 platform, following the manufacturer’s recommendations.

### 2.5 Data analysis

The paired-end reads obtained by sequencing were initially assigned to their respective samples based on unique barcodes. Barcode and primer sequences were removed by truncation. Subsequently, the paired-end reads were merged using the Fast Length Adjustment of SHort reads (FLASH) tool (Magoc and Salzberg, 2011). To ensure high-quality data, quality filtering was performed on the raw reads to obtain clean tags using fqtrim (v0.9.7) (Pertea, 2018). Chimeric sequences were filtered using VSEARCH software (v2.3.4) (Rognes et al., 2016). Following data preprocessing, dereplication was performed using DADA2 (Callahan et al., 2016), resulting in the generation of an amplicon sequencing variant (ASV) table and ASV sequences. The abundance of ASVs was normalized to the relative abundance of each sample based on the SILVA (release 138, https://www.arb-silva.de/documentation/release138/) and NT-16S database (release 20230718). Alpha and beta diversity analyses were conducted to assess microbial diversity within and between samples. Alpha diversity, which measures the complexity of microbial diversity within each sample, was assessed using the Shannon and Simpson indices after rarefaction. Beta diversity, which evaluates differences in microbial diversity between samples, was analyzed based on weighted UniFrac metrics (Lozupone et al., 2011) using principal coordinate analysis (PCoA) and cluster analysis after the data were rarefied, and examined the significance using Adonis test as reported previously (Anderson, 2001). The betadisper analysis was also performed using ANOVA test to further determine the difference in spread of samples between two groups as described previously (Anderson, 2006). These analyses were performed using QIIME2 software (Bolyen et al., 2019), and the resulting graphs were generated using the R package. To assess the statistical significance of bacterial abundance and diversity among different groups of endometrial samples, Mann–Whitney U test was used, and Benjamini-Hochberg false discovery rate (FDR)-adjusted p-value (Q-value) was used to correct for multiple comparisons. Linear discriminant effect size (LEfSe) analysis was used to determine significant differences in species composition and community results (Segata et al., 2011), and Linear discriminant analysis (LDA) was used to assess the effect size of each feature (LDA score (log 10) = 3 as the cut-off value). Phylogenetic Investigation of Communities by Reconstruction of Unobserved States (PICRUSt2) v2.2.0b was used to predict the potential metagenome functionality based on 16S ASV content for functional analysis (Douglas et al., 2020). The ASV table and corresponding representative sequences were aligned (NSTI cut-off value of 2) to a reference phylogenetic tree, and the software predicted functional gene families and copy numbers for each specific ASV. The resulting output generated an abundance profile of pathways based on the KEGG database (Kanehisa and Goto, 2000). Differential pathways between the groups were identified and presented using STAMP software (v2.1.3) (Parks et al., 2014) with t-test. Benjamini-Hochberg FDR-adjusted p values <0.05 were considered significant.

### 2.6 ASV-based biomarker identification and classifier model construction

All recruited patients from the EPs and control groups were randomly divided into the discovery and validation cohorts. In the discovery phase, the genera ASVs which were significantly enriched in the endometrial samples of patients with EPs detected using Mann–Whitney U tests were selected. Identification was performed on an advanced random forest model (R version 4.1.3) using the ASV abundance profile of the discovery cohort as described previously (Ren et al., 2019), depending on both the mean decrease accuracy and the mean decrease Gini. The optimal ASV-based biomarkers were then selected and calculated to predict the probability of a sample being in the EP or control groups in the discovery cohort. In the validation phase, the diagnostic value of selected ASV-based biomarkers was investigated again in the validation cohort. Receiver operating characteristic (ROC) curves were obtained (SPSS version 20.0) to evaluate the constructed models, and the area under the ROC curve (AUC) was used to evaluate the accuracy of the models.

### 2.7 Statistical analysis

Associations between clinical characteristics were assessed using Student t test for continuous covariables, and Fisher’s exact test or Pearson’s chi-square test, depending on the nature of the variables. Two-tailed p-values were calculated, and a Benjamini-Hochberg FDR adjustment was applied to account for multiple comparisons. Statistical significance was set at p < 0.05. All statistical analyses were conducted using SPSS software version 20 (IBM). Visualizations and diagrams were generated using the R package version 3.5.2.

## 3 Results

### 3.1 Study design and patient information

**Figure 1:**
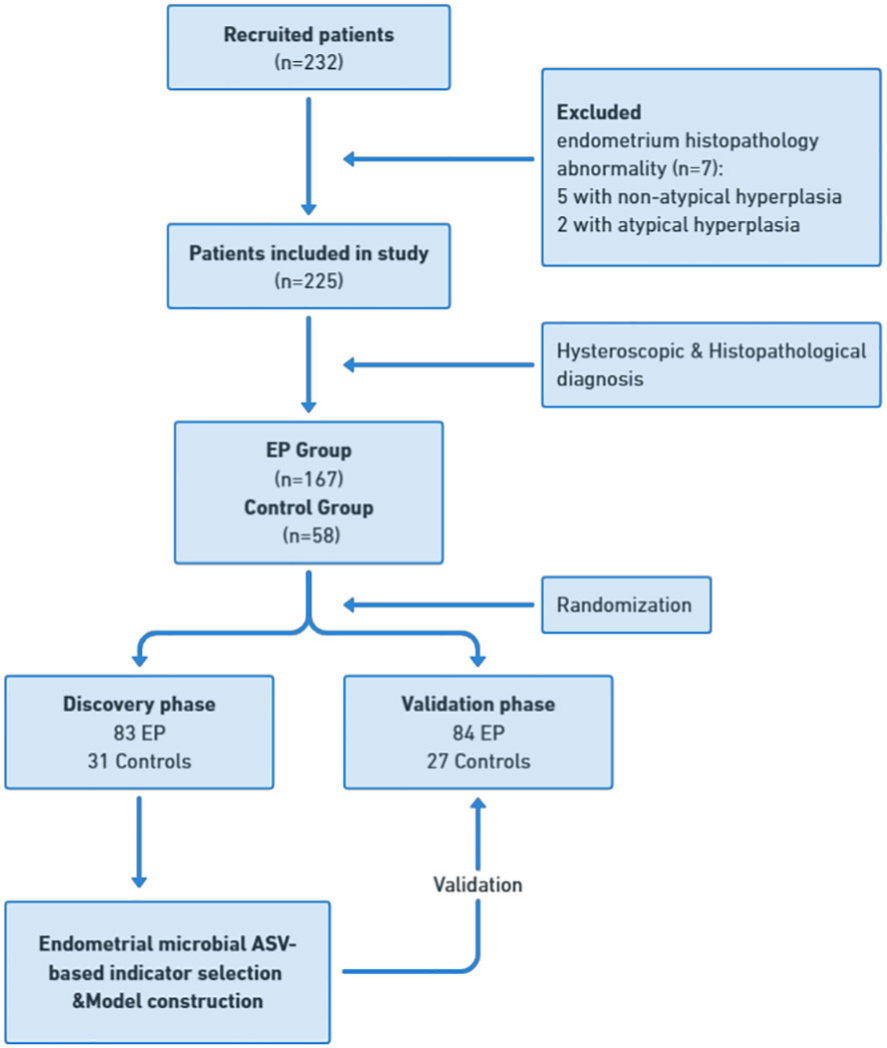
Study design and patient flow diagram. Endometrial samples were prospectively collected from 232 recruited patients undergoing hysteroscopy. After applying exclusion criteria, including abnormal endometrium histopathology, a cohort of 225 patients was established for final analysis. This cohort was divided into 167 patients in the EP group and 58 in the control group, with further subdivision into discovery and validation cohorts. In the discovery phase, endometrial microbial ASV-based indicators were identified in the discovery cohort with 83 EP and 31 controls, leading to the construction of an EP classifier. In the validation phase, this classifier’s diagnostic efficacy was tested in the validation cohort with 84 EP patients and 27 controls. EP: endometrial polyp.

A total of 232 patients who met the inclusion criteria were enrolled in this study. Among them, five patients were excluded due to the presence of atypical hyperplasia, and two patients were excluded due to atypical hyperplasia on endometrial histology. In total, 225 patients were included in the final analysis. Based on the combined hysteroscopy and histopathological diagnosis, 167 patients were categorized into the EP group, whereas 58 patients were placed in the control group (**Figure 1**). In the control group, there were 32 cases of intrauterine adhesions (55.2%), 17 cases of cervical polyps (29.3%), 6 cases of submucosal fibroids (10.3%), and one case each of incomplete abortion, infertility, and uterine septum (1.7%). The clinical characteristics of the two groups are shown in **Table 1**. The proportion of nulliparous patients in the EP group was significantly lower than in the control group (p = 0.008), whereas the proportion of patients with AUB was significantly higher in the EP group than in the control group (p < 0.001). The positive rate of CD138 IHC staining in the EP group was significantly higher than that in the control group (p < 0.001). However, there were no significant differences between the two groups with respect to age, body mass index (BMI), parity, history of cesarean section, or the proportion of patients with elevated glycosylated hemoglobin levels.

**Table 1:** Clinical characteristics of the enrolled patients.

Characteristics	EP(n=167)	Control(n=58)	P value
Age (mean ± SD)	35.83 ± 7.06	35.41 ± 8.71	0.715^#^
BMI (mean ± SD) kg/m^2^	21.60 ± 3.01	21.38 ± 2.98	0.640^#^
Gravid (times)			0.148^*^
0 ≥1	43 (25.7%) 124 (74.3%)	9 (15.5%) 49 (84.5%)	
Parity (times)			0.008^*^
0 ≥1	53 (31.7%) 114 (68.3%)	30 (51.7%) 28 (48.3%)	
Cesarean section history	46 (27.5%)	10 (17.2%)	0.158^*^
Infertility	9 (5.4%)	1 (1.7%)	0.459^*^
AUB symptom	108 (64.7%)	12 (20.7%)	<0.001^*^
Menostaxis Intermenstrual bleeding Menorrhagia Metrorrhagia	34 (31.5%) 57 (52.8%) 6 (5.6%) 11 (10.2%)	2 (16.7%) 2 (16.7%) 0 (0%) 8 (66.7%)	
HbA1c proportion (%) ≥6.0 <6.0	12 (7.2%) 155 (92.8%)	5 (8.6%) 53 (91.4%)	0.773^*^
Positive CD138 IHC staining	76 (45.5%)	10 (17.2%)	<0.001^*^
EP counts			
Single Multiple	47 (28.1%) 120 (71.9%)		
Previous history of EP surgery	21 (12.6%)		

Data is presented as mean ± standard deviation or n (%).

# Student t test.

*Pearson’s chi-squared test.

EP, endometrial polyp; BMI, body mass index; AUB, abnormal uterine bleeding.

### 3.2 Bacterial diversity of the endometrial microbiota

**Figure 2:**
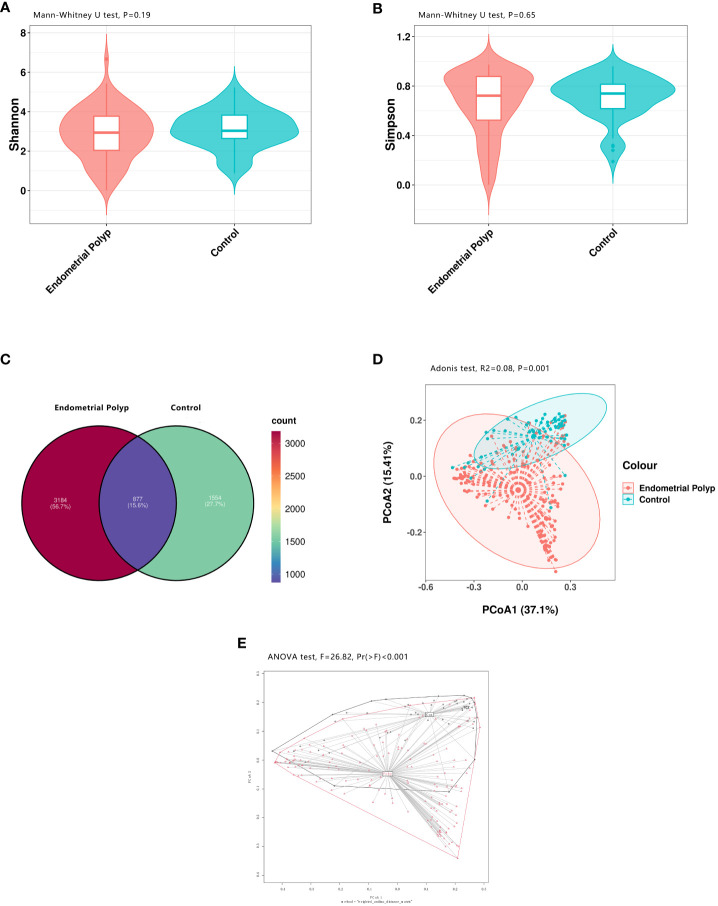
Bacterial diversity in the endometrial microbiota. Both alpha and beta diversity measures were compared between the EP and control groups. The endometrial microbial diversity was estimated using the Shannon index (p = 0.19) **(A)** and the Simpson index (p = 0.65) **(B)** with Mann-Whitney U test. A Venn diagram **(C)** illustrates the overlap of 877 shared ASVs between the two groups. Beta diversity, calculated using principal coordinate analysis (PCoA, weighted UniFrac, Adonis test), shows a significant difference in endometrial microbial community composition between the EP and control groups (p = 0.001, R^2^ = 0.08)) **(D)**. The betadisper analysis also shows significant difference in spread of samples between the EP and control groups (F=26.82, p<0.001) with ANOVA test **(E)**. Polyp: Endometrial polyp, Con: Control.

High-throughput 16S rRNA gene sequencing was performed based on 225 endometrial samples (167 from patients with EP and 58 from control) to examine the endometrial microbial community composition. The minimum number of 16S sequences read was 10,291, and the maximum was 79,563. The average number of sequences read for all study samples was 49,326 ± 18,998. Species richness and evenness within the populations were assessed using Shannon and Simpson indices based on the sample ASVs. The analysis revealed no significant differences in the endometrial microbial communities between the EP and control groups (**Figures 2A, B**). The Venn diagram illustrates that there were 877 shared ASVs between the EP and control groups. Additionally, the EP group had 3184 unique ASVs, accounting for 56.7% of the total, whereas the control group had 1554 unique ASVs, accounting for 27.7% of the total (**Figure 2C**). To visually represent the microbiota space between individuals, we performed PCoA based on the weighted UniFrac distance matrix with significance analysis by Adonis test. The results revealed a distinct difference in the distribution of the endometrial microbial community composition between the two groups (p = 0.001, R^2^ = 0.08, **Figure 2D**). For further determination, the betadisper analysis was performed and a significant difference in spread of samples between the EP and control groups was found (F=26.82, p<0.001, **Figure 2E**).

### 3.3 Endometrial microbiota composition associated with EPs

**Figure 3:**
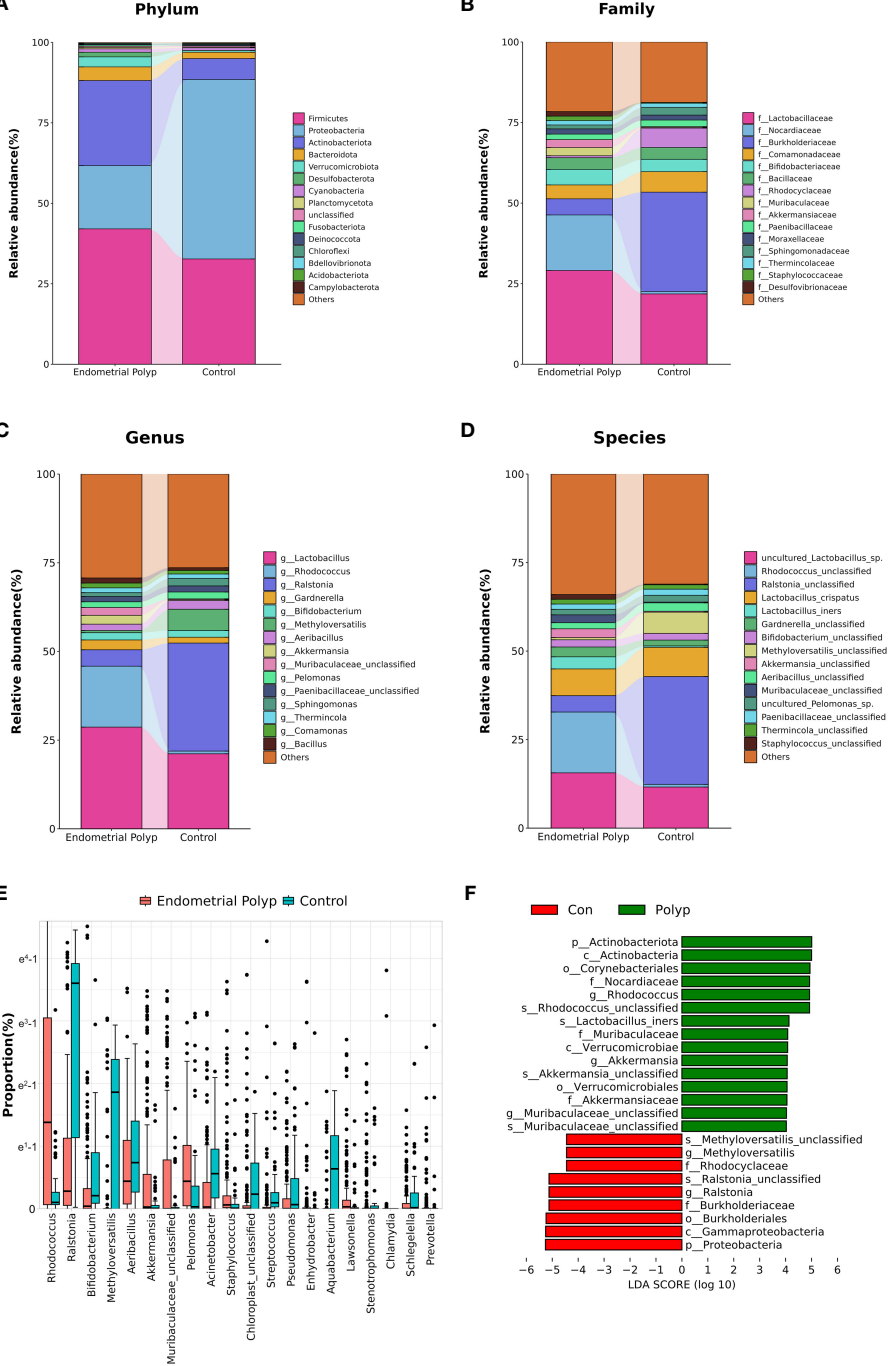
Endometrial microbiota composition. The average composition of the bacterial community at the phylum **(A)**, family **(B)**, genus **(C)**, and species **(D)** levels. Panel **(E)** highlights the top 20 amplicon sequencing variants (ASVs) showing significant differences at the genus level between EP and control groups according to proportion using Mann-Whitney U test with Benjamini-Hochberg FDR-adjusted p-value <0.05. Linear Discrimination Analysis (LDA) **(F)** of differential abundance at the genus level demonstrates the effect size of genera with altered abundance in the two groups using by Mann-Whitney U test with Benjamini-Hochberg FDR-adjusted p-value <0.05 and LDA score (log 10) > 3. Polyp: Endometrial polyp, Con: Control.

The composition of the top 15 taxa of the endometrial microbiota at the phylum, family, genus, and species levels, based on their relative abundances, is shown in **Figures 3A–D**. In the EP group, the five dominant phyla were Firmicutes, Actinobacteriota, Proteobacteria, Bacteroidota, and Verrucomicrobiota, whereas those in the control group included Proteobacteria, Firmicutes, Actinobacteriota, Bacteroidota, and Cyanobacteria. The five dominant genera in the endometrial polyp group were *Lactobacillus*, *Rhodococcus*, *Ralstonia*, *Gardnerella*, and *Akkermansia*, whereas those in the control group were *Ralstonia*, *Lactobacillus*, *Methyloversatilis*, *Aeribacillus*, and *Sphingomonas*. We conducted differential abundance analysis to identify significant changes in the dominant endometrial microbiota at the phylum, genus, and species levels between the two groups; the top 10 microorganisms in relative abundance at each level are shown in **Tables 2**–**4**. The top 20 genera with significant changes, according to their relative proportions, are shown in **Figure 3E**. LDA demonstrated that the abundances of *Rhodococcus*, *Akkermansia* and *Muribaculaceae_unclassified* were significantly higher in the EP group than in the control group, with an Benjamini-Hochberg FDR adjusted p-value <0.05 and LDA score >3. Conversely, the abundance of *Ralstonia* and *Methyloversatilis*, were significantly lower in the EP group than in the control group (**Figure 3F**). Furthermore, at the species level, the abundance of *Lactobacillus iners* was significantly higher in the EP group than that in the control group (**Figures 3D, F** and **Table 4**).

**Table 2:** Composition of endometrial microbiota at the phylum level.

Taxonomy	Mean proportion	Control (n=58)	Q value
	EP (n=167)		
Firmicutes	42.06% (36.90% - 47.10%)	32.71% (25.85% - 39.86%)	0.38
Proteobacteria	19.64% (16.45% - 22.89%)	55.74% (49.15% - 61.94%)	<0.001
Actinobacteriota	26.45% (22.04% - 31.08%)	6.52% (4.47% - 9.19%)	<0.001
Bacteroidota	4.24% (3.01% - 5.51%)	1.96% (1.24% - 2.91%)	0.64
Verrucomicrobiota	3.11% (2.04% - 4.21%)	0.52% (0.27% - 0.83%)	0.11
Desulfobacterota	1.44% (0.63% - 2.44%)	0.04% (0.02% - 0.07%)	0.39
Cyanobacteria	1.06% (0.43% - 1.91%)	0.84% (0.58% - 1.16%)	<0.001
Planctomycetota	0.46% (0.15% - 0.89%)	0.21% (0.06% - 0.40%)	0.003
Unclassified_Bacteria	0.36% (0.25% - 0.49%)	0.39% (0.23% - 0.57%)	0.77
Fusobacteriota	0.40% (0.03% - 1.03%)	0.08% (0.05% - 0.11%)	<0.001

This table presents the mean proportions of the ten most abundant microorganisms at the phylum level in both the EP group and the control group. Differential analysis was conducted using the Mann–Whitney U test with Benjamini-Hochberg FDR-adjusted p-value (Q value). Additionally, 95% bootstrap percentile confidence intervals are denoted within parentheses.

**Table 3:** Composition of endometrial microbiota at the genus level.

Taxonomy	Mean proportion	Control (n=58)	Q value
	EP (n=167)		
*Lactobacillus*	28.67% (23.76% - 34.38%)	21.15% (14.49% - 28.68%)	0.65
*Rhodococcus*	17.17% (13.16% - 21.46%)	0.75% (0.25% - 1.62%)	<0.001
*Ralstonia*	4.63% (2.85% - 6.59%)	30.46% (24.20% - 36.37%)	<0.001
*Gardnerella*	2.77% (1.43% - 4.58%)	1.65% (0.26% - 3.50%)	0.67
*Bifidobacterium*	2.05% (0.55% - 3.81%)	1.90% (0.80% - 3.43%)	<0.001
*Methyloversatilis*	0.61% (0.24% - 1.04%)	5.99% (4.50% - 7.42%)	<0.001
*Aeribacillus*	1.79% (1.23% - 2.52%)	2.46% (1.66% - 3.47%)	0.02
*Akkermansia*	2.49% (1.60% - 3.44%)	0.26% (0.08% - 0.49%)	0.003
*Muribaculaceae_unclassified*	2.22% (1.49% - 3.08%)	0.21% (0.06% - 0.40%)	0.14
*Pelomonas*	1.60% (1.21% - 2.02%)	1.93% (0.80% - 3.54%)	<0.001

This table presents the mean proportions of the ten most abundant microorganisms at the genera level in both the EP group and the control group. Differential analysis was conducted using the Mann–Whitney U test with Benjamini-Hochberg FDR-adjusted p-value (Q value). Additionally, 95% bootstrap percentile confidence intervals are denoted within parentheses.

**Table 4:** Composition of endometrial microbiota at the species level.

Taxonomy	Mean proportion	Control (n=58)	Q value
	EP (n=167)		
*uncultured_Lactobacillus_sp.*	15.62% (11.71% - 19.64%)	11.61% (6.29% - 18.31%)	0.83
*Rhodococcus_unclassified*	17.17% (13.27% - 21.61%)	0.75% (0.26% - 1.68%)	<0.001
*Ralstonia_unclassified*	4.63% (2.85% - 6.81%)	30.46% (23.85% - 36.40%)	<0.001
*Lactobacillus_crispatus*	7.55% (5.10% - 10.39%)	8.24% (3.55% - 13.84%)	0.23
*Lactobacillus_iners*	3.42% (1.73% - 5.52%)	0.40% (0.01% - 0.96%)	0.004
*Gardnerella_unclassified*	2.77% (1.35% - 4.55%)	1.65% (0.23% - 3.51%)	0.66
*Bifidobacterium_unclassified*	2.04% (0.57% - 3.92%)	1.90% (0.76% - 3.68%)	0.001
*Methyloversatilis_unclassified*	0.61% (0.24% - 1.08%)	5.99% (4.49% - 7.42%)	<0.001
*Akkermansia_unclassified*	2.49% (1.60% - 3.48%)	0.26% (0.07% - 0.452%)	0.004
*Aeribacillus_unclassified*	1.72% (1.22% - 2.35%)	2.37% (1.61% - 3.28%)	0.04

This table presents the mean proportions of the ten most abundant microorganisms at the species level in both the EP group and the control group. Differential analysis was conducted using the Mann–Whitney U test with Benjamini-Hochberg FDR-adjusted p-value (Q value). Additionally, 95% bootstrap percentile confidence intervals are denoted within parentheses.

### 3.4 Predictive functional analysis of endometrial microbiota associated with EPs

**Figure 4:**
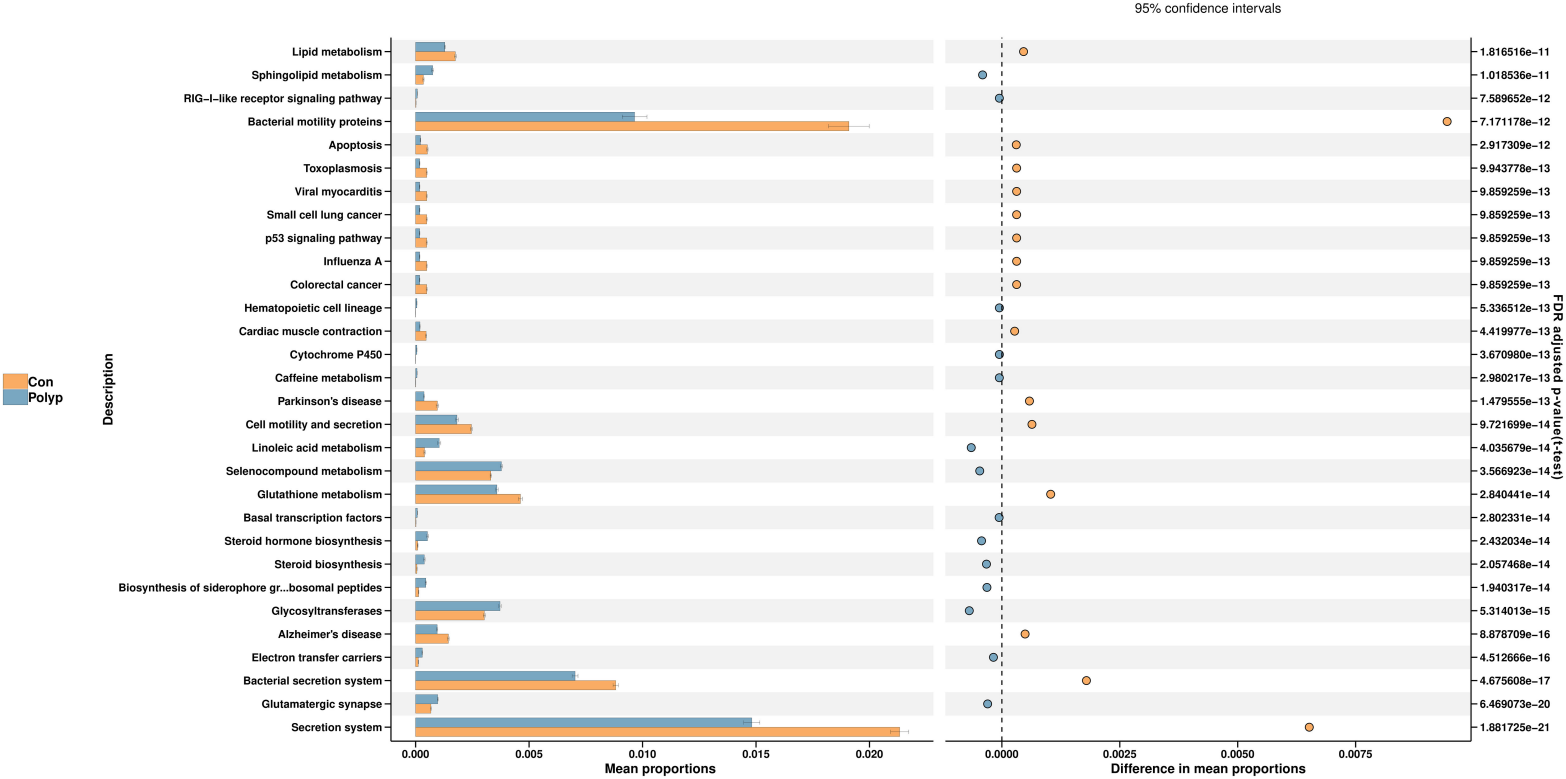
Predicted microbial metabolism pathways in the endometrial microbiota of the EP and control groups. PICRUSt2 was utilized to analyze the differential KEGG pathways. The left bar represents the mean proportion of the KEGG pathways, whereas the right-hand side figure illustrates the difference in mean proportions of pathways enriched in each group using t-test and Benjamini-Hochberg FDR-adjusted p-value. Polyp: Endometrial polyp, Con: Control.

In this study, we employed the Phylogenetic Investigation of Communities by Reconstruction of Unobserved States 2 (PICRUSt2) to predict functional changes in the endometrial microbiota. The PICRUSt2 analysis revealed several potential pathway alterations in the microbial communities between the EP and control groups, particularly associated with metabolism and cellular processes. Specifically, the EP group exhibited a significant enrichment of pathways related to sphingolipid metabolism, linoleic acid metabolism, selenocompound metabolism, steroid hormone biosynthesis, and glycosyltransferases. Conversely, pathways related to apoptosis, glutathione metabolism, motility, and secretion were significantly lower in the EP group than in the control group (**Figure 4**).

### 3.5 Identification of endometrial microbial ASV-based indicators for EPs

**Figure 5:**
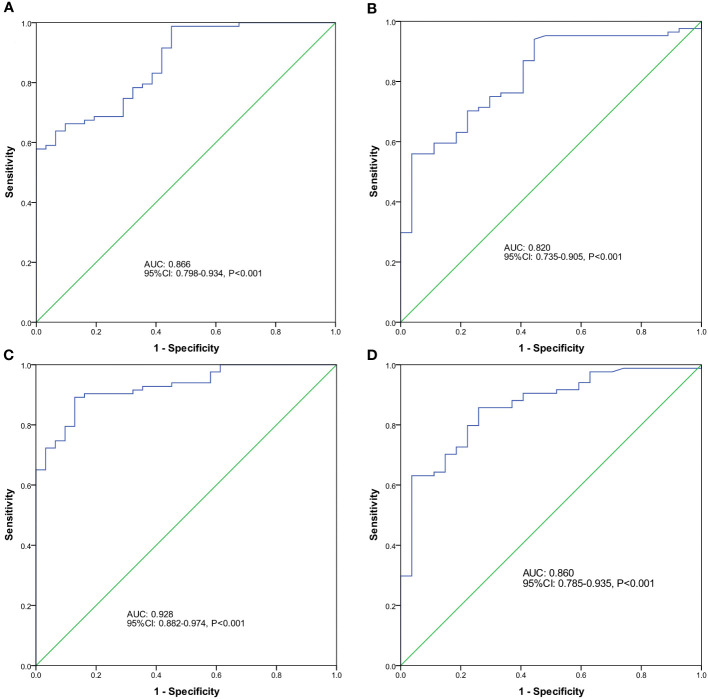
Predictive potential of endometrial microbial amplicon sequencing variant (ASV)-based indicators for EP. The receiver operator characteristic (ROC) curve analysis was performed to assess the predictive ability of different factors for EP using optimal ASV-based biomarkers identified by random forest models in the discovery cohort **(A)** and validation cohort **(B)**, as well as the ROC curves for ASVs in combination with the presence of abnormal uterine bleeding (AUB) symptoms in the discovery **(C)** and validation **(D)** cohorts.

To further explore the diagnostic value of endometrial microbiota alterations for EP, we constructed a classification model that could specifically distinguish between the EP and non- hyperproliferative endometrium groups. All enrolled patients were randomly allocated into two cohorts. The discovery cohort comprised 83 cases of EP and 31 controls, while the validation cohort consisted of 84 cases of EP and 27 controls. In the discovery phase, 86 ASVs were significantly enriched in the EP group based on random forest model analysis in the discovery cohort (**Table S1**). As a result, *Rhodococcus*, *Bifidobacterium*, *Streptomyces*, *Chloroplast_unclassified* and *Akkermansia* were selected as 5 optimal ASV-based indicators to construct a classifier model based on an integrated ranking related to the mean decrease accuracy and mean decrease Gini (**Table S2**). The model reached an AUC value of 86.6% (95% CI: 79.8–93.4%) (**Figure 5A**) in the discovery cohort. In the validation phase (data from the validation cohort), the classifier model reached an AUC value of 82.0% (95% CI: 73.5–90.5%) (**Figure 5B**).Furthermore, when combining the ASV-based markers with the presence of AUB symptoms, the AUC value further improved to 92.8% (95% CI: 88.2–97.4%, **Figure 5C**) in the discovery cohort and 86.0% (95% CI: 78.5–93.5%, **Figure 5D**) in the validation cohort.

## 4 Discussion

The dynamic balance and stability of the genital tract microbiota are vital for maintaining women’s reproductive health. Studies utilizing second-generation sequencing technology have indicated that changes in the genital tract microbiota are linked to various reproductive system disorders, such as cervical intraepithelial neoplasia, endometrial hyperplasia, endometriosis, and endometrial cancer. However, the specific mechanisms and influencing factors of EPs are still not fully understood, and the interaction between the endometrium and microbiota requires further investigation. In the present study, we aimed to explore the microecological characteristics of EPs in the uterine cavity using the largest sample size to date. Our objective was to delineate the community structure and potential function of the endometrial microbiota in patients with EP and to further identify diagnostic indicators for EPs based on specific endometrial microbial ASVs.

First, we reviewed the clinical characteristics of both patient groups. Among women of reproductive age, AUB is the most common symptom associated with EPs (Munro et al., 2011). In our study, we observed a significantly higher incidence of AUB in the EP group than in the control group (64.7% vs. 20.7%, p < 0.001). Intermenstrual bleeding (52.8%) and menostaxis (31.5%) were the predominant bleeding patterns, which is consistent with previous research findings (Golan et al., 2001). Previous studies have also highlighted the close connection between the EPs and CE (Cicinelli et al., 2019; Vitagliano et al., 2021; Qu et al., 2023). Consistent with the work of Cicinelli (Cicinelli et al., 2019), our study found a significantly higher proportion of CE in the EP group than in the control group (45.5% vs. 17.2%, p < 0.001). This confirms that inflammation is a significant marker of EP formation.

The composition of the microbiota in the female genital tract can be influenced by various factors, including hormone levels, nutritional status, pregnancy, and glucose-lipid metabolism (Koedooder et al., 2019; Molina et al., 2020). Therefore, in our investigation, we carefully compared baseline differences between patients with EPs and controls with respect to these factors. Our findings revealed that the proportion of nulliparous women was significantly lower in the EP group than that in the control group. This difference could be attributed to the high proportion of patients with intrauterine adhesions in the control group, which may affect fertility. However, it is noteworthy that there were no significant differences between the two groups in terms of age, BMI, parity, history of a cesarean section, or blood glucose status. Bleeding is a significant contributing factor to changes in microbial composition, and abnormal uterine bleeding is closely associated with endometrial polyps. Therefore, we hypothesize that abnormal uterine bleeding-induced alterations in the abundance and diversity of endometrial microbiota would essentially mirror the microbial characteristics associated with endometrial polyps. These results allowing us to objectively assess and discern any variations in the endometrial microbiota that may be associated with EPs.

We evaluated the diversity of endometrial microbiota in the EP and control groups and found no significant differences in Shannon and Simpson indices, indicating that the overall microbial community diversity was similar between the two groups. However, beta diversity analysis revealed significant differences, suggesting that the EP group had a distinct microbial composition compared to the control group. To further analyze the differences in inter-group distribution, we also conducted betadisper test. The result continues to show significant disparities, indicating that some factors beyond species composition may influence the distribution between the two groups simultaneously. Fang et al. reported that patients with EPs exhibited a higher Shannon diversity index and a larger number of phylotypes than infertile patients without EPs (Fang et al., 2016). Conversely, Liang et al. found no significant differences in alpha and beta diversities between the two groups (Liang et al., 2023). The disparities in these results could be attributed to variations in the selection of the control group. In our study, the control group comprised patients with non- hyperproliferative endometrial diseases, leading to a relatively complex disease composition and large variations in the group, which may contribute to the differences observed in species composition between the groups. In contrast, the control groups in the aforementioned studies consisted of patients with infertility without other uterine factors, resulting in a more homogeneous disease composition and smaller variations.

Previous studies have extensively explored alterations in the uterine cavity microbiota of patients with EPs, including an elevated proportion of Firmicutes and a decreased proportion of Proteobacteria at the phylum level. Furthermore, enrichment of specific genera such as *Lactobacillus*, *Staphylococcus*, *Bifidobacterium*, *Gardnerella*, and *Bacteroides* was observed, along with a decreased level of *Pseudomonas* at genus levels (Fang et al., 2016; Horban et al., 2019; Liang et al., 2023). Our study aligns with these previous findings and confirms significant abundance changes in the endometrial microbiota of patients with EPs, particularly in relation to *Proteobacteria*, *Bifidobacterium*, *Staphylococcus*, and *Pseudomonas*. These results provide strong evidence of microbial dysbiosis in the uterine cavity of patients with EPs. Additionally, our study identified an increased proportion of the phylum Actinobacteriota as well as changes in the abundance of several traditional reproductive tract pathogens at the genus level, including an increase in *Chlamydia* and a decrease in *Streptococcus* and *Prevotella*. These novel observations suggest that traditional pathogens may be involved in the development of EPs.

In our study, we made a novel discovery regarding the significant enrichment of *Rhodococcus* in the endometrial microecology of patients with EPs. Interestingly, the presence of *Rhodococcus* in the female reproductive tract microbiota has rarely been reported in previous EP studies. Chao et al. reported a higher abundance of *Rhodococcus* in the uterine lavage fluid samples of patients with endometrial hyperplasia and endometrial cancer than in those with benign endometrial lesions, suggesting a potential association with abnormal endometrial proliferation (Chao et al., 2022). Similarly, Lu et al. found an enrichment of *Rhodococcus* in the uterine cavity of patients undergoing hysterectomy for endometrial cancer and other benign diseases, although the difference in abundance between the two groups was not statistically significant (Lu et al., 2021). Additionally, Zhao et al. reported a lower abundance of *Rhodococcus* in the vaginal microbiota of patients with recurrent miscarriages than in the control group, indicating a potential association with recurrent spontaneous abortion (RSA) (Zhao et al., 2021). However, direct evidence that *Rhodococcus* causes female reproductive tract-related diseases is lacking. *Rhodococcus* has a phenotype between *Mycobacterium* and *Norcardia* and is primarily distributed in the environment. Certain species, such as *Rhodococcus equi*, can cause opportunistic infections in immunocompromised individuals, leading to pneumonia, sepsis, and systemic multi-organ infections (Weinstock and Brown, 2002). Our discovery of the abnormal enrichment of *Rhodococcus* in the uterine cavity of patients with EPs adds a new dimension to our understanding of the role of the uterine microbiota in gynecological health. Further research is required to investigate the potential pathogenic mechanisms and clinical implications of *Rhodococcus* in EPs and other reproductive tract disorders.

The composition of the uterine cavity microbiota has been debated, with studies yielding varying results. Although it is widely believed to have a low bacterial load in a highly diverse environment (Chen et al., 2017; Laniewski et al., 2020), the dominant microbial population in the uterine cavity remains a topic of discussion. Most studies on endometrial diseases have shown that *Lactobacillus* is predominant in most patients (Fang et al., 2016; Lozano et al., 2021; Chen et al., 2023; Liang et al., 2023). However, Winters found *Acinetobacter* and *Pseudomonas* were predominant in the uterine cavity, with low levels of *Lactobacillus* (Winters et al., 2019). In our study of patients with EPs, *Lactobacillus* remained the dominant bacterial group in the endometrium, while it was not dominant in the control group. *Lactobacillus* was previously thought to maintain vaginal microbial homeostasis through the production of hydrogen peroxide and lactic acid to inhibit the growth of other bacteria; however, its actual distribution and role in the uterine cavity remain controversial. Interestingly, the species-level analysis revealed that the abundance of *Lactobacillus iners* was significantly higher in the EP group than in the control group. *Lactobacillus iners* has been re-evaluated in recent years and is distinct from other common vaginal *Lactobacilli* (such as *Lactobacillus crispatus*) in its overall protective effect on the vaginal microbiota. Under certain conditions, it acts as an opportunistic pathogen and is associated with bacterial vaginosis, sexually transmitted diseases, and adverse pregnancy outcomes (Zheng et al., 2021; Bloom et al., 2022). Considering this, we speculate that abnormal colonization and increased abundance of *Lactobacillus iners* in the uterine cavity of patients with EP may contribute to changes in the endometrial microbiota balance and participate in the formation of EPs through microbe-host interactions. However, it is essential to further validate and explore these differences using more accurate techniques, such as metagenomics, owing to the limitations of the 16S rRNA gene sequencing technology for species-level detection. Such investigations will help delineate the role of specific bacterial species in EPs and their potential impact on women’s reproductive health.

Next, we conducted a predictive functional analysis of the microbial community using PICRUSt2 and found that pathways related to sphingolipid metabolism and steroid hormone biosynthesis were significantly upregulated in the EP group. Sphingolipid metabolism is involved in the regulation of cell proliferation, and its altered activity has been associated with conditions such as endometriosis and endometrial cancer (Knapp et al., 2010; Lee et al., 2014). Studies in rat models have confirmed their roles in the proliferation of uterine epithelial cells (Cerbon et al., 2018). This disruption may contribute to abnormal cell proliferation and ultimately associate with the formation of EPs in the endometrium. Furthermore, the significant increase in pathways related to steroid hormone biosynthesis observed in EPs may also relates with local imbalances in estrogen and progesterone activities in the endometrium, which have been identified as crucial mechanisms in the development of EPs (Kossai and Penault-Llorca, 2020).

Based on the findings outlined above, we hypothesized that specific changes in the endometrial microbiota composition may contribute to EP formation. Specific species like *Lactobacillus iners*, may act as opportunistic pathogens, and can induce local inflammation or damage by affecting pathways including sphingolipid metabolism and apoptosis. Moreover, these alterations may influence the synthesis and metabolism of steroid hormones within the endometrial microenvironment, thus playing a role in the development or progression of EPs. However, further investigations are needed to confirm these hypotheses and fully understand the complex interactions between the endometrial microbiota, sphingolipid metabolism, and steroid hormone biosynthesis in the context of EPs.

Finally, we identified specific endometrial microbial markers that could effectively differentiate patients with EPs from those with normal or benign non- hyperproliferative endometrial diseases. The combined classification model based on the optimal microbial ASV-based indicators along with the presence of AUB symptoms showed high accuracy in distinguishing between the two groups, both in the discovery and validation cohorts. Considering the significant false-positive rate of ultrasound scanning in distinguishing between pathological polyps and functional polyps or endometrial polypoid changes, biomarkers derived from the endometrial microbiota may provide a more precise approach to diagnosis and treatment planning. Indeed, the rapid, convenient, and minimally invasive nature of endometrial sampling makes it suitable for implementation in outpatient settings. This diagnostic and predictive technique has the potential to effectively prevent unnecessary hysteroscopic surgeries, thereby reducing medical risks and healthcare costs, particularly in regions with limited access to office hysteroscopy.

## 5 Limitations

Due to the invasive nature of endometrial sampling, we were unable to include healthy women without any clinical symptoms as a control group due to ethical considerations. The control group consisted of patients with non- hyperproliferative endometrial diseases, such as intrauterine adhesions and submucosal fibroids, who were scheduled for hysteroscopy. While this approach provides valuable insights, it may introduce certain biases in the comparison of endometrial microbial characteristics between the two groups, owing to the absence of completely healthy individuals as negative controls. Additionally, the limited sample size and nature of the study prevented us from conducting further analyses of infertility, recurrence, and other clinical implications associated with EPs. Moreover, the study population predominantly consisted of Asian individuals, highlighting the need for additional research to confirm the generalizability of these findings to different racial and geographical populations. Furthermore, the 16S rRNA gene sequencing technology has limitations, primarily focusing on microbial changes at the genus level. Consequently, the accuracy of detecting species-level microbiota and predicting functional outcomes may be limited. To overcome these limitations, further validation and exploration are required using advanced techniques, such as metagenomics and metabolomics, which can provide more detailed and comprehensive insights into the uterine microbial environment. Overall, although this study yielded significant findings regarding the endometrial microbiota and its association with EPs, acknowledging these limitations is crucial for accurately interpreting the results and guiding future research efforts to enhance our understanding of this complex area of study.

## 6 Conclusions

Overall, our study revealed that significant endometrial microbial dysbiosis is linked to changes in specific metabolic pathways in patients with EPs. This suggests that alterations in endometrial microecology may play a pivotal role in disease development. A predictive model based on endometrial microbial indicators and clinical symptoms holds promise as a precise and efficient method of diagnosing EPs. Further investigations are likely to yield valuable insights into the underlying mechanisms, leading to improved approaches for targeted therapeutic interventions and preventive strategies.

## Data availability statement

The datasets presented in this study can be found in online repositories (https://www.ncbi.nlm.nih.gov/bioproject/PRJNA1004537/). The names of the repository/repositories and accession number(s) can be found below: NCBI PRJNA1004537.

## Ethics statement

The studies involving humans were approved by The Ethics Committee of the Women’s Hospital, School of Medicine, Zhejiang University (IRB-20210280-R). The studies were conducted in accordance with the local legislation and institutional requirements. The participants provided their written informed consent to participate in this study.

## Author contributions

YZ: Formal analysis, Funding acquisition, Investigation, Methodology, Writing – original draft. YL: Formal analysis, Funding acquisition, Investigation, Methodology, Writing – original draft. GX: Formal analysis, Investigation, Software, Supervision, Writing – review & editing. YW: Conceptualization, Funding acquisition, Project administration, Resources, Supervision, Writing – review & editing.
