# Supplementary material for: Endometrial microbiota alteration in female patients with endometrial polyps based on 16S rRNA gene sequencing analysis
Source: Front Cell Infect Microbiol. 2024 Apr 9;14:1351329. doi: 10.3389/fcimb.2024.1351329 (PMC11035718; doi:10.3389/fcimb.2024.1351329)
Supplement: Supplementary file 1 [file Table_1.docx]

Table S1. ASVs identified with significantly enrichment in the patients with EP based on random forest model analysis in the discovery phase. EP, endometrial polyp.

Table S2. Five optimal ASV markers selected in the discovery phase between the EP group and the control group. EP, endometrial polyp.
